# Supplementary material for: WordCommentsAnalyzer: A windows software tool for qualitative research
Source: F1000Res. 2018 Sep 4;7:536. Originally published 2018 May 3. [Version 2] doi: 10.12688/f1000research.14819.2 (PMC6137411; doi:10.12688/f1000research.14819.2)
Supplement: Supplementary file 3 [file f1000research-7-17614-s0002.tgz › b2a79496-4ada-47c4-9682-a99ce867836d.zip!/Eight Iranian Health Journals/Journal of Education and Community Health_6.docx]

© Journal of Education and Community Health. This work is licensed under CC BY-NC 4.0 (https://creativecommons.org/licenses/by-nc/4.0/).

Volume & Issue: دوره 3 - شماره 3

Title: بررسی انجام خودآزمایی پستان و عوامل تاثیرگذار بر آن بر اساس نظریه رفتار برنامه ریزی شده در زنان شهر کرمان

http://jech.umsha.ac.ir/article-1-242-fa.html

سابقه و هدف: سرطان پستان شایع‌ترین نوع بدخیمی در زنان است که تشخیص به موقع آن در درمان موفقیت آمیز بیماری تأثیر بسزایی دارد. هدف از این مطالعه بررسی انجام خودآزمایی پستان و عوامل تأثیرگذار بر آن بر اساس نظریه رفتار برنامه ریزی شده در زنان شهر کرمان بود. مواد و روش‌ها: پژوهش حاضر یک مطالعه توصیفی-تحلیلی از نوع مقطعی بود که بر روی 300 نفر از زنان مراجعه کننده به مراکز بهداشتی درمانی شهر کرمان انجام شد. روش نمونه گیری بصورت خوشه‌ای و در دسترس بود. ابزار اندازه گیری پرسشنامه‌ای مشتمل بر اطلاعات جمعیت شناختی، متغیر آگاهی و سازه‌های نظریه رفتار برنامه ریزی شده بود. داده‌ها توسط نسخه 21 نرم افزار SPSS و با استفاده از آزمون‌های همبستگی پیرسون، اسپیرمن، کای اسکوئر، رگرسیون لجستیک، تی‌مستقل و من‌ویتنی مورد تجزیه و تحلیل قرار گرفت. یافته‌ها: میانگین سن شرکت کنندگان در مطالعه 8.18±30.26 بود. 12.7 درصد زنان بطور ماهیانه و مرتب معاینه پستان را انجام می‌دادند. مهمترین علت عدم انجام خودآزمایی پستان فقدان مهارت در این زمینه گزارش گردید. آگاهی مهمترین پیش‌بینی کننده انجام خودآزمایی پستان بود (0.05>OR=0.896 ,P). میانگین نمرات آگاهی، نگرش، کنترل رفتاری درک شده و قصد رفتاری زنان دارای سابقه انجام خودآزمایی با زنان بدون سابقه انجام خودآزمایی اختلاف معنی‌داری داشت (0.05>P). نتیجه‌گیری: مداخلات آموزشی با بهره‌گیری از نظریه رفتار برنامه‌ریزی شده در جهت ارتقاء سطح آگاهی و توانمند سازی زنان در تشخیص و درمان به موقع سرطان پستان اجرا گردد.

Title: بررسی تأثیر آموزش مهارت‌های ارتباطی در زنان مراجعه کننده به مراکز بهداشتی درمانی شهر بهار با استفاده از تئوری حمایت اجتماعی در افزایش رضایتمندی زناشویی زوجین

http://jech.umsha.ac.ir/article-1-237-fa.html

سابقه و هدف: ارتباط زناشویی به عنوان عمیق‏ترین نوع ارتباط همواره مورد توجه انسان بوده است. این مطالعه با هدف تعیین تأثیر آموزش مهارت‌های ارتباطی بر افزایش رضایتمندی زناشویی زوجین در زنان مراجعه کننده به مراکز بهداشتی درمانی شهر بهار بر مبنای تئوری حمایت اجتماعی انجام شد. مواد و روش‌ها: این مطالعه تجربی در بین 100 نفر از زنان مراجعه کننده به مراکز بهداشتی درمانی شهر بهار در استان همدان در سال 1395 انجام گردید. جهت نمونه گیری، ابتدا از ۳ مرکز بهداشتی درمانی شهر بهار 100 نفر به طور تصادفی انتخاب و در ادامه شرکت کنندگان به تصادف به دو گروه آزمون و کنترل (هر گروه 50 نفر) تقسیم شدند. ابزار جمع­آوری اطلاعات شامل سه پرسشنامه رضایتمندی زناشویی Enrich، پرسشنامه حمایت اجتماعی و پرسشنامه مهارتهای ارتباطی Burton بود که به روش خودگزارش دهی توسط زوجین تکمیل گردید. 3 جلسه آموزشی و 2 جلسه مشاوره در طی زمان 2 هفته تنها برای گروه آزمون برگزار و به فاصله یکماه پرسشنامه های پس آزمون تکمیل گردید. داده‌ها با استفاده از آزمون‌های آماری تی‌مستقل، تی‌زوجی، آزمون دقیق فیشر و آنالیز کوواریانس در نسخه 16 نرم افزار SPSS تحلیل گردید. یافته‌ها: میانگین نمرات مهارتهای ارتباطی، رضایت زناشویی و حمایت اجتماعی گروه آزمون نسبت به گروه کنترل در مرحله پس از آزمون به طور معناداری افزایش یافته بود (0.05>P). نتیجه‌گیری: آموزش مهارت‌های ارتباطی می‌تواند تأثیر بسزایی در ارتقاء رضایتمندی زناشویی داشته باشد. یکی از مهمترین علت‌های آن، حمایت‌های اجتماعی توسط خانواده می‌باشد، لذا می‌توان از آن به عنوان یک رویکرد مداخله‌ای مؤثر استفاده نمود.

Title: بررسی تأثیرمداخله آموزشی بر ارتقاء رفتارهای سالم تغذیه‌ای در دانش آموزان مقطع ابتدایی شهر کرمانشاه

http://jech.umsha.ac.ir/article-1-223-fa.html

سابقه و هدف: موضوع تغذیه در کودکان و سنین بالندگی علاوه بر نقش سلامت ساز و حیات بخش خود، می‌تواند در بهبود جریان رشد و پیشگیری از بروز عفونت‌ها مؤثر باشد. لذا، این مطالعه با هدف بررسی تأثیر مداخله آموزشی بر ارتقای رفتارهای سالم تغذیه‌ای در دانش آموزان مقطع ابتدایی شهر کرمانشاه در سال 95 انجام گردید. مواد و روش­ ها: پژوهش حاضر یک مطالعه نیمه تجربی بود که تعداد 135 دانش آموز مقطع ابتدایی از طریق نمونه گیری تصادفی در دو گروه آزمون و کنترل در آن شرکت کردند. اطلاعات مطالعه حاضر توسط یک پرسشنامه روا و پایا در دو مرحله قبل و بعد از مداخله در حیطه‌های آگاهی، نگرش، عملکرد و متغیرهای جمعیتی جمع‌آوری شد. داده‌ها توسط نسخه 13 نرم افزار آماری SPSS و با استفاده از آزمون آماری تی زوجی مورد تجزیه و تحلیل قرار گرفت. یافته­ ها: نتایج مطالعه در خصوص مقایسه نمرات متغیرهای آگاهی، نگرش و عملکرد گروه آزمون قبل و بعد از مداخله آموزشی حاکی از افزایش معنی‌دار نمرات بود (0.001>P)، ولیکن اختلاف معنی‌داری در این نمرات قبل و بعد از مداخله گروه کنترل مشاهده نشد (0.05<P). نتیجه‌گیری: نتایج مطالعه نشان داد که مداخله آموزش موجب تغییر مثبت و فزآینده بر آگاهی، نگرش و به ویژه رفتار دانش آموزان در موضوع تغذیه سالم شده است. با توجه به بستر مناسب آموزش در مدرسه و کم هزینه و اثربخش بودن آموزش تغذیه‌ای، لزوم تعمیم این گونه برنامه‌های آموزشی ضروری به نظر می‌رسد.

Title: مقایسه میزان استفاده از بازی‌های رایانه‌ای و پرخاشگری در دانش‌آموزان پسر مقطع راهنمایی شهر تهران

http://jech.umsha.ac.ir/article-1-230-fa.html

سابقه و هدف: فن­ آوری نوین، اوقات زیادی از فرصت‌های نوجوانان را پر می­ کند. برنامه­ های این رسانه­ ها، حاوی الگوهای فرهنگی و اخلاقی ویژه ­ای است که می­ تواند بر روی نوجوانان تأثیر فوق­ العاده­ ای داشته باشد. پژوهش حاضر با هدف مقایسه میزان استفاده از بازی‌های رایانه‌ای و پرخاشگری در دانش­آموزان پسر مقطع راهنمایی شهر تهران صورت پذیرفت. مواد و روش‌ها: پژوهش حاضر یک مطالعه توصیفی بود که جامعه‌ آماری پژوهش را تمامی دانش­ آموزان پسر مقطع راهنمایی شهر تهران که در سال تحصیلی 95-94 مشغول به تحصیل بودند، تشکیل می­ دادند. از میان جامعه آماری 120 دانش‌آموز پسر که 60 نفر رفتار پرخاشگری و وابستگی به رایانه و 60 نفر نیز دارای رفتار عادی و عدم وابستگی به رایانه بودند به روش تصادفی چندمرحله ­ای از مناطق (شمال، جنوب، شرق و غرب) تهران انتخاب شدند. برای جمع‌آوری اطلاعات از پرسشنامه پرخاشگری (AGQ) و پرسشنامه محقق ساخته شامل 10 سؤال چندگزینه‌ای که استفاده یا عدم استفاده از بازی ­های رایانه ­ای را می­ سنجید، استفاده گردید. داده ­ها وارد نسخه 19 نرم‌افزار SPSS شده و با استفاده از آزمون‌های آماری ضریب همبستگی پیرسون و تی­مستقل تجزیه و تحلیل شدند. یافته‌ها: نتایج پژوهش نشان داد که بین انجام بازی­ های رایانه­ ای و پرخاشگری دانش ­آموزان و همچنین بین مدت زمان استفاده از بازی­ های رایانه­ ای و میزان پرخاشگری رابطه معنی‌داری وجود دارد (0.05>P). نتیجه‌گیری: با توجه به یافته‌های پژوهش، به نظر می­رسد که با مدیریت و کنترل مدت زمان و نوع استفاده از بازی‌های رایانه‌ای می‌توان از اثرات تخریبی این رسانه کاست.

Title: بررسی سواد سلامت و عوامل مؤثر بر آن در دانشجویان خوابگاهی دانشگاه علوم پزشکی شهید بهشتی تهران

http://jech.umsha.ac.ir/article-1-218-fa.html

سابقه و هدف: سواد سلامت نشان دهنده مهارت‌های شناختی و اجتماعی است که انگیزه و توانایی افراد برای کسب، دسترسی و درک روش‌های استفاده از اطلاعات جهت حفظ و ارتقاء سلامتی را تعیین می‌کند. این مطالعه با هدف سنجش میزان سواد سلامت در دانشجویان و شناسایی عوامل مؤثر بر آن در  سال تحصیلی 95-94 انجام گرفت. مواد و روش‌‌ها: مطالعه توصیفی-تحلیلی به روش مقطعی بر روی 360 نفر از دانشجویان خوابگاهی دانشگاه علوم پزشکی شهید بهشتی تهران که از طریق نمونه گیری خوشه‌ای تصادفی انتخاب شده بودند، انجام گردید. داده‌ها با استفاده از پرسشنامه سنجش سواد سلامت جمعیت شهری ایرانی (HELIA) جمع‌آوری گردید. داده‌های به دست آمده به کمک نسخه 21 نرم افزار SPSS و با به کارگیری آمارهای توصیفی و آزمون کای دو تحلیل شدند. یافته‌ها: میانگین و انحراف معیار سن دانشجویان 4.05±22.93سال بود. 9.2 درصد (31 نفر) سواد سلامت ناکافی، 27.6درصد (94 نفر) سواد سلامت نه چندان ناکافی، 42.6 درصد (145 نفر) سواد سلامت کافی و 19.7 درصد (67 نفر) سواد سلامت عالی داشتند. سواد سلامت ارتباط معنی‌داری با جنسیت و درآمد ماهیانه خانواده دانشجویان داشت (P<0.001). نتیجه گیری: با توجه به محدود بودن سواد سلامت در تعداد زیادی از دانشجویان، لازم است برنامه‌های آموزشی متناسب با سطح سواد سلامت و علائق رسانه‌ای آنان، طراحی شوند.

Title: بررسی وضعیت عملکرد مراقبت از پا و چشم در بیماران دیابتی نوع دو در شهر فسا: کاربرد نظریه رفتار برنامه ریزی شده

http://jech.umsha.ac.ir/article-1-254-fa.html

سابقه و هدف: زخم پای دیابتی و مشکلات چشمی از جمله شایع‌ترین عوارض بیماری دیابت محسوب می‌شوند که با مراقبت و کنترل می‌توان از بروز یا ناتوانی ناشی از آن جلوگیری نمود. مطالعه حاضر با هدف بررسی وضعیت مراقبت از پا و چشم در بیماران دیابتی نوع دو  براساس نظریه  برنامه‌ریزی شده در  شهر فسا  انجام گرفت. مواد و روش‌ها: در این مطالعه مقطعی 151 نفر از بیماران مبتلا به دیابت نوع 2 مراجعه کننده به کلینیک دیابت بیمارستان شریعتی فسا بصورت تصادفی ساده در سال 1395 انتخاب و وارد مطالعه شدند. داده ها با استفاده از پرسشنامه‌ای مشتمل بر اطلاعات جمعیت شناختی و سنجش سازه‌های نظریه رفتار برنامه‌ریزی شده (نگرش، هنجار انتزاعی، کنترل رفتاری درک شده و قصد رفتاری) و عملکرد مراقبت از پا و چشم جمع آوری شد. داده­ ها وارد نسخه 20 نرم افزار SPSS شده و با استفاده از آزمون‌های آماری ضریب همبستگی پیرسون و آنالیز رگرسیون خطی تجزیه و تحلیل شدند. یافته‌ها: میانگین سنی افراد مورد مطالعه 12.4±52.17 سال بود. میزان آگاهی و عملکرد مراقبت از پا و چشم در بین بیماران در سطح پایین و میزان نگرش، هنجار انتزاعی و قصد افراد در سطح متوسط ارزیابی گردید. سازه‌های آگاهی، نگرش، کنترل رفتاری درک شده، هنجار انتزاعی و قصد رفتاری پیش بینی کننده عملکرد مراقبت از پا و چشم در بیماران مبتلا به دیابت نوع 2 بودند و بطور کلی سازه‌های مورد بررسی 31.6 درصد از عملکرد مراقبت از پا و 28.4 درصد از عملکرد مراقبت از چشم را پیشگویی می‌نمودند. نتیجه‌گیری: بر اساس نتایج این مطالعه، طراحی و اجرای برنامه‌های آموزشی نظریه محور بر ارتقاء عملکرد مراقبت از پا و چشم در بیماران دیابتی پیشنهاد می‌گردد.

Title: بررسی رفتارهای تغذیه ای سالمندان مراجعه کننده به مراکز بهداشتی درمانی شهر ایلام

http://jech.umsha.ac.ir/article-1-167-fa.html

زمینه و هدف: افزایش روز افزون جمعیت سالمندان و گسترش بیماری ­های دوران سالمندی ضرورت توجه به سلامت این قشر از جامعه را دو چندان می­ کند.  تغذیه یکی از تعیین­ کننده­ های عمده در پیشگیری از بیماری­ های مزمن دوران سالمندی است. هدف از انجام مطالعه حاضر تعیین رفتارهای تغذیه ­ای سالمندان شهر ایلام می‌باشد. مواد و روش­ ها: تعداد 200 نفر از سالمندان بالای 60 سال با میانگین سنی 2.37±61.15 به روش نمونه­ گیری در دسترس در این مطالعه مقطعی وارد شدند. داده­ ها با استفاده از پرسش­نامه محقق ساخته که شامل اطلاعات جمعیت­ شناختی و پرسشنامه رفتارهای تغذیه­ ای سالمندان بود، بصورت خودگزارشی گردآوری شد. روایی پرسشنامه با نظر پانل خبرگان و پایایی آن با محاسبه آلفای کرونباخ تأیید گردید. داده ها با استفاده از نسخه 19 نرم افزار SPSS و آزمون ­های همبستگی پیرسون، تی‌مستقل و آنالیز واریانس یک­طرفه مورد تجزیه و تحلیل قرار گرفت. یافته­ ها: افراد مورد مطالعه به­ طور میانگین نمره 5.46±32.59 از دامنه نمره قابل اکتساب عملکرد تغذیه­ ای بین 10 تا 40 را کسب کردند. نتایج مطالعه حاضر نشان داد که بین گروه­ های جنسی، تحصیلی و همین­طور شغلی از نظر انجام رفتارهای تغذیه­ ای اختلاف معنی ­داری وجود ندارد (r= 0.215, P = 0.002). همچنین مقادیر مصرف میوه و سبزیجات، حبوبات، انواع گوشت و شیر و لبنیات ناکافی گزارش گردید. نتیجه ­گیری: نتایج مطالعه حاضر نشان دهنده دریافت ناکافی گروه‌های مهم غذایی در سالمندان از جمله میوه و سبزیجات، حبوبات و لبنیات بود. بنابراین پیشنهاد می ­شود برنامه­ ریزی لازم جهت ارتقاء رفتارهای تغذیه ­ای سالمندان توسط مسئولان و کارشناسان سلامت صورت گیرد.
